# Lessons learned exploiting a multi‐year large‐scale data set derived from operational quality assessment of mosquito larval treatments in rain catch basins

**DOI:** 10.1002/ps.70016

**Published:** 2025-06-23

**Authors:** Chiara Virgillito, Eleonora Longo, Silvia Paolucci, Martina Micocci, Mattia Manica, Federico Filipponi, Stefano Vettore, Davide Bonetto, Andrea Drago, Simone Martini, Alessandra Della Torre, Beniamino Caputo

**Affiliations:** ^1^ Department of Public Health and Infectious Diseases Sapienza Università di Roma Rome Italy; ^2^ Center Agriculture Food Environment University of Trento San Michele all'Adige Italy; ^3^ Center for Health Emergencies Fondazione Bruno Kessler Trento Italy; ^4^ National Research Council – Institute for Environmental Geology and Geoengineering (CNR‐IGAG) Montelibretti Italy; ^5^ Entostudio srl Ponte San Nicolò Italy

**Keywords:** larvicide, mosquito, quality control, catch basins

## Abstract

**BACKGROUND:**

Larval control is crucial for managing mosquito arbovirus vectors. Quality control (QC) data from insecticide‐based interventions are rarely exploited to assess the effectiveness of biocides in the field. This study aims to: (i) evaluate the outcomes of large‐scale catch basin treatments with diflubenzuron (DFB) and *Bacillus thuringiensis* var. *israelensis* and *B*. (*Lysinibacillus*) *sphaericus* (Bti + Bs) on immature stages of *Aedes albopictus* and *Culex pipiens*, with reference to critical variables, such as the intervals between catch basin treatments and inspections; and (ii) identify areas of repeated treatment failures.

**RESULTS:**

More than 30 000 catch basins were inspected from 2019 to 2021 for mosquito immature stages after treatment with DFB and Bti + Bs in 461 municipalities in north‐east Italy. Overall, 5% of catch basins revealed the presence of live L3–L4 larvae and/or pupae. Model results showed opposite associations between percentages of positive catch basins treated with the two larvicides and the intervals between treatments and inspections, likely because of the different modes of action; i.e., a negative association following DFB treatments (day 7/day 21: *Ae. Albopictus*, 7%–4%; *Cx*. *pipiens*, 8%–4%), and a positive association following Bti + Bs treatments for *Ae. albopictus* (day 7/day 21: 2%–13%). Spatial analysis revealed repeated DFB treatment failures against *Cx. pipiens* in the area of the Venice lagoon, where the highest frequencies of alleles associated with DFB resistance have been reported.

**CONCLUSION:**

The results show that, despite unavoidable limitations, high‐quality area‐wide databases from multi‐year QC activities of public mosquito control interventions may allow general conclusions to be reached (such as shortening the intervals between Bti + Bs treatments) accounting for real‐world heterogeneities that cannot be achieved experimentally. © 2025 The Author(s). *Pest Management Science* published by John Wiley & Sons Ltd on behalf of Society of Chemical Industry.

## Graphical abstract

Results highlight opposite associations between percentages of positive rain catch basins treated with diflubenzuron and *Bacillus* against mosquito larvae and/or pupae and the intervals between treatments and inspections, and spatial variability in vector control.

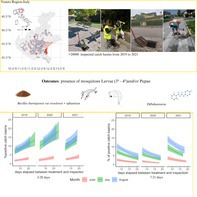

## 1 INTRODUCTION

The invasive mosquito *Aedes albopictus* and the native species *Culex pipiens* are the primary vectors of arboviruses in Europe. The incidence of these diseases in Europe has surged recently, with *Ae. albopictus* transmitting dengue (DENV) and chikungunya (CHIKV), and *Cx. pipiens* transmitting West Nile virus and Usutu virus.^1^, ^2^, ^3^ In North‐east Italy, West Nile virus has become endemic since 2008 with an increasing number of human cases in past years (https://www.epicentro.iss.it//) and several autochthonous cases/outbreaks of *Aedes‐*transmitted arbovirus have been reported since 2007 (CHIKV^4^; DENV^5^, ^6^, ^7^). The recurrent occurrence of autochthonous cases of arboviruses recorded in Italy in recent years underscores the urgent need for effective mosquito control interventions to reduce the risk of transmission to humans.

According to European Centre for Disease Prevention and Control guidelines^8^ mosquito larval control is the primary preventive measure to reduce the risk of arbovirus outbreaks in Europe, owing to its low impact on non‐target species.^9^, ^10^ Adulticide spraying is recommended to be restricted only in cases of arbovirus autochthonous transmission because of the high environmental impact and the need to reduce the development of resistant populations and preserve adulticide effectiveness. Cost‐effectiveness analyses indicate that area‐wide calendar‐based larval control in public areas significantly outperforms the costs associated with disease treatment and hospitalization due to *Aedes*‐transmitted diseases,^11^ making it a convenient investment for municipalities. Only a limited number of larvicides are currently approved for use in mosquito larval control in the European Union (EU)^12^: (i) bacteria, such as toxins from *Bacillus thuringiensis* var. *israelensis* and *B*. (*Lysinibacillus*) *sphaericus* (Bti + Bs); or (ii) chemicals, such as insect growth regulators (IGR) [e.g. diflubenzuron (DFB), *S*‐methoprene and pyriproxyfen]^13^, ^14^ (https://www3.epa.gov/pesticides/), and more recently, larvicides that act as physical barriers, such as polydimethylsiloxane. Biocide authorization can change rapidly in the EU, for example in the case of the SC‐15 product family based on DFB (e.g., DU‐DIM SC‐15, Europe PMC no. 19161 and device SC‐15, Europe PMC no. 19033), which from 2025 will no longer be allowed for mosquito control.^12^

In Italy, mosquito larval control in public areas in urban and peri‐urban sites mostly takes the form of calendar‐based larvicide interventions, very frequently carried out by municipalities to reduce nuisance and the risk of arbovirus transmission by *Cx. pipiens* and *Ae. albopictus*. These interventions require scheduling and funding by municipalities and public health institutions, implementation by competent operators, and quality assurance (QA) as well as quality control (QC) processes carried out third‐party experts to assess transparency in treatment planning and implementation, and their outcomes, respectively.^15^ In practice, several municipalities – particularly in north‐east regions and in touristic coastal areas – allocate resources for calendar‐based treatments for larval control and issue a public procurement tender that details the authorized active ingredients, their application methods, the treatment frequency, and the larval breeding sites to be treated. The latter are usually represented by rain catch basins, which are largely colonized by *Cx. pipiens* and *Ae. albopictus* from April to October,^16^ despite the two species displaying a slightly different temporal dynamic, with *Cx. pipiens* prevailing in spring and autumn^17^ and *Ae. albopictus* prevailing in the summer months.^18^

Although several studies have assessed the efficacy of larvicidal products in controlled laboratory and field environments,^19^, ^20^, ^21^, ^22^, ^23^ to our knowledge no studies have tried to assess the outcomes of calendar‐based area‐wide interventions taking into account real‐world factors, such as eco‐climatic conditions and species‐specific susceptibility to larvicides. This is because data from QC are usually recorded only in the grey literature (thereby limiting their accessibility to the scientific community), and their exploitation is complicated by large eco‐climatic variability and a lack of control sites.

This paper reports the main lessons learned exploiting a multi‐year large‐scale data set derived from operational QC assessment of routine mosquito larval control interventions implemented in the Veneto region (North‐east Italy) from 2019 to 2021, with the goal of assessing the outcomes of catch basin treatments on *Ae. albopictus* and *Cx. pipiens* immature stages. The results allow us to go beyond the customary quality assessment at the local level and to reach general conclusions accounting for real‐world heterogeneities.

## 2 MATERIAL AND METHODS

### 2.1 Sampling area

The Veneto region in north‐eastern Italy covers an area of 18 399 km^2^ with 4 879 133 inhabitants in 2020, distributed across 560 municipalities; it has a population density of 266 inhabitants/km^2^ (Supporting Information, Fig. S1(A)). The landscape is predominantly flat (Padan Plain, ~56%); mountainous areas cover 29%, and hills account for 15%. The study area mostly includes municipalities in the Padan Plain and the neighbouring hills. Supporting Information, Fig. S2 shows the average daily temperature and rainfall during the study period (June–August).

### 2.2 Implementation of larval control in public areas

In 2019, 2020 and 2021, larvicidal treatments were systematically planned by Veneto municipalities based on a regional plan^24^ published yearly by the Veneto region (https://bur.regione.veneto.it/). This regional plan was drafted in accordance with the National Plan for Arbovirosis.^25^ The plan prescribed larvicide treatments in catch basins within public areas to be executed by tendered private pest control companies between April and October. The larvicidal formulations authorized were either bacterial‐based formulations (Bti + Bs), or IGRs (DFB, pyriproxyfen and *S*‐methoprene), or silicon‐based formulations (polydimethylsiloxane, only in Venice since 2020). The recommended dose for catch basins adhered to the product's technical data sheet. A Bti + Bs formulation required applications every 4–6 weeks, whereas the other products required applications every 3–4 weeks (https://echa.europa.eu/). Treatments were undertaken by 37 and 45 pest control companies in 2020 and 2021, respectively; no data were available for 2019. For QA purposes, the pest control companies were required to report specific details of each treatment, including the product used, the treatment schedule and the number and location of catch basins treated. For QC purposes, third‐party technicians authorized by the region were requested to inspect 30 randomly chosen catch basins per municipality to verify the outcome of the treatment by regional guidelines.^24^ If the number of positive catch basins per municipality was <10%, a portion of the expenses incurred by municipalities for treatment implementation was reimbursed by the region.

### 2.3 Quality control

QC was carried out by technicians and experts (Entostudio s.r.l.) in 500 municipalities; that is, all Veneto municipalities except the 60 in the province of Belluno (Supporting Information, Fig. S1(B), Table S1). Inspections were carried out between 2 and 28 days after treatment based on the expected effectiveness of the larvicidal formulation concerning and taking into account environmental factors (e.g. rain). The outcome of the larvicide treatments was assessed by three consecutive dippings of 500 mL of water per catch basin for a total of ~30 treated catch basins per municipality, regardless of the total number of catch basins per municipality. The following data were recorded: date of inspection, presence of water, and presence, species and stage of collected mosquitoes. A catch basin was classified as ‘negative’ when there was sufficient water for three dippings and no mosquito larvae of L3–L4 instars or pupae were collected. The inspected catch basins had various shapes and sizes: the most common type was a square structure (~ 45 cm long × 45 cm wide × 50–100 cm deep) covered with a metal grid and often equipped with a drainage pipe ~10 cm deep, allowing excess rainwater to drain away.

### 2.4 Climate variables

Spatially explicit daily cumulative rainfall (CRF) maps were generated to provide each inspected catch basin with the corresponding rainfall time series. First, geographic coordinates of the treated catch basins were identified using the geocode function available in the ‘ggmap’ R library.^26^ Daily CRF records from the 191 weather stations available to ARPA Veneto were acquired for the period 2019–2021, georeferenced and spatially interpolated into a raster grid with a 120‐m cell size using the thin plate spline with regularization algorithm (parameterized as follows: smoothing factor = 0.1; point cloud thinning factor = 1.5). Finally, the daily CRF for each inspected catch basin was set by spatial query of the daily CRF spatial maps on the geographical coordinates of the treatment sites.

Data were collected using R version 4.2.2,^27^ while spatial interpolation and query were done using GRASS GIS.^28^

### 2.5 Statistical analysis

The outcome of the most used larvicide treatments was evaluated using a generalized linear model (GLM) assuming a Bernoulli distribution for the response variable; that is, L3, L4 and/or P presence or absence. Inspections were carried out between 7 and 21 days after DFB treatments^29^ and between 2 and 28 days after Bti + Bs treatment.^23^, ^30^ We developed separate models for *Ae. albopictus* in DBF‐treated catch basins (GLM‐1), *Cx. pipiens* in DBF‐treated catch basins (GLM‐2), *Ae. albopictus* in Bti + Bs‐treated catch basins (GLM‐3) and *Cx. pipiens* in Bti + Bs‐treated catch basins (GLM‐4).

All models have the following covariates: number of days elapsed between treatment and inspection (hereafter DTI quantitative variable); CRF calculated as the sum of daily CRF during the period between treatment and inspection (quantitative variable); months of inspection (qualitative variable: June/July/August); years of inspection (qualitative variable: 2019/2020/2021).

Assessment of the statistical assumptions of GLMs was carried out by inspecting Pearson residuals $r_{i}=\frac{\left(O_{i}-E_{i}\right)}{\surd E_{i}}$, where $O_{i}$ is the observed percentage of *i‐*positive catch basins and $E_{i}$ is the estimated percentage of *i‐*positive catch basins. To investigate potential spatial patterns in residuals, the mean residuals of the catch basin were computed for each municipality and year. Municipalities in which the mean residuals exceeded the 90th percentile of the residual distribution allowed us to identify those in which the GLMs, as informed by the selected covariates, underestimated the occurrence of L3, L4 and pupae (P) of *Ae. albopictus* and *Cx. pipiens*. This analysis may help identify municipalities in which the presence of mosquitoes after treatment is not expected yet detected.

Finally, we performed two sensitivity analyses. First, we considered only catch basins that contained at least L1 and/or L2 instar larvae of *Ae. albopictus* and *Cx. pipiens*; that is, where ongoing mosquito activity was confirmed. Second, we considered as the response variable the number of positive catch basins (using the same biological infestation at the individual catch basin level – DBF: L3, L4 and P between 7 and 21 days; Bti + Bs: L3, L4 and/or P between 2 and 28 days) out of the total catch basins in each municipality and assumed a binomial distribution in the GLMs. We considered the mean DTI and CRF values for each municipality.

Descriptive statistics were carried out using $\chi^{2}$ and Student's *t*‐test. All analyses were run with R version 4.3.0.^27^

## 3 RESULTS

On average 84% of the municipalities (2019: *n* = 306 of 363; 2020: *n* = 345 of 353; 2021: *n* = 281 of 323) appropriately declared the larvicide product used and treatment dates. The most used larvicides were DFB (2019: 60%; 2020: 66%; 2021: 50%) and Bti + Bs (2019: 28%; 2020: 33%; 2021: 40%). Approximately 48% of the municipalities (222 of 461) were inspected in each of the three years (Supporting Information, Table S1).

In total, 39 841 catch basins in 461 municipalities were inspected to assess the presence of mosquito larvae (L) and/or pupae (P) (Supporting Information, Fig. S1(B), Table S2). These were treated with: DFB (56%, *n* = 22 426) either in tablet or liquid form; Bti + Bs (28%, *n* = 11 344) mostly in a granular formulation; pyriproxyfen (1%, *n* = 637); *S*‐methoprene (*n* = 107); and polydimethylsiloxane (*n* = 61, only in Venice since 2020) (Supporting Information, Table S2).

During QC inspections, 11% of catch basins were dry (2019: 15%, *n* = 2086; 2020: 17%, *n* = 2241; 2021: 1%, *n* = 129) and were excluded from the analyses. Overall, 18 385 catch basins inspected between day 7 and day 21 after DFB treatment, and 9940 catch basins inspected between day 2 and day 28 after Bti + Bs treatment were included in the analyses (Table 1).

**Table 1:** Inspected catch basins in the Veneto region treated with either diflubenzuron (DFB) or *Bacillus thuringiensis* var. *israelensis + Bacillus sphaericus* (Bti + Bs) during 2019, 2020 and 2021

	2019		2020		2021		Overall	
	DFB	Bti+Bs	DFB	Bti+Bs	DFB	Bti+Bs	DFB	Bti+Bs
Inspected catch basins (*n*)	5873	2665	6574	2842	5938	4433	18385	9940
Municipalities (*n*)	203	98	227	117	160	130	333	208
Catch basins positive for mosquitoes (%; 95%CI)	8.6 (7.9, 9.3)	8.4 (7.4‐9.5)	8.8 (8.1, 9.5)	14.2 (13.1‐15.6)	8.2 (7.5, 8.9)	7.6 (6.9‐8.4)	8.5 (8.1‐8.9)	9.9 (9.3‐10.5)
Catch basins positive for *Ae. albopictus* (%; 95%CI)	5 (4.5, 5.6)	5.9 (5.1, 6.9)	4.2 (3.7, 4.7)	8.8 (7.8‐9.9)	5.2 (4.6, 5.7)	4.7 (4.1‐5.4)	4.8 (4.5‐5.1)	5.8 (5.3‐6.2)
Catch basins positive for *Cx. pipiens* (%; 95%CI)	5.8 (5.2, 6.4)	5.1 (4.3, 6.0)	6.7 (6.1, 7.3)	8.7 (7.7, 9.8)	4.6 (4.1, 5.1)	4.1 (3,5, 4.7)	5.7 (5.4‐6.1)	6.3 (5.8‐6.8)
Days between treatment and inspection (median)	11	10	12	12	10	11	11	11

*Note*: All the characteristics/indexes were calculated between 7 and 21 days after treatment with DFB and between 2 and 28 days after treatment with Bti + Bs. Positive, presence of mosquito L3 + L4 larvae and/or pupae (*Anopheles*, *Culiseta*, *Aedes* and *Culex*) according to the active substance; DFB, presence of pupae; Bti + Bs, presence of larvae L3 and/or L4 and/or pupae; CI, confidence interval.

### 3.1 Outcomes of DFB treatments at catch basin level

Among the 18 385 catch basins inspected between 7 and 21 days after DFB treatment, 8.5% contained mosquito L3, L4 and P (4.8% *Ae. albopictus* and 5.7% *Cx. pipiens*) (Table 1). No statistical differences are observed among the three years in the percentage of catch basins positive for mosquito L3, L4, P ($\chi^{2}$ =0.02, *P* > 0.05), or *Ae. albopictus* ($\chi^{2}$ = 0.10, *P* > 0.05), or *Cx. pipiens* ($\chi^{2}$ =0.40, *P* > 0.05) (Table 1). The monthly observed percentage of DFB‐treated catch basins with alive *Ae. albopictus* L3, L4 and P have a similar pattern each year with a low value in May (<1%), a progressive increase up to a peak in August (9%) and a decrease in September (3%) (Supporting Information, Fig. S2 (left)). Only in 2020, is an opposite trend observed for the percentage of DFB‐treated catch basins with alive *Cx. pipiens* pupae, with the highest value in May (14%) and a decrease in the following months (<1% in September) (Supporting Information, Fig. S3 (left)).

**Figure 1:**
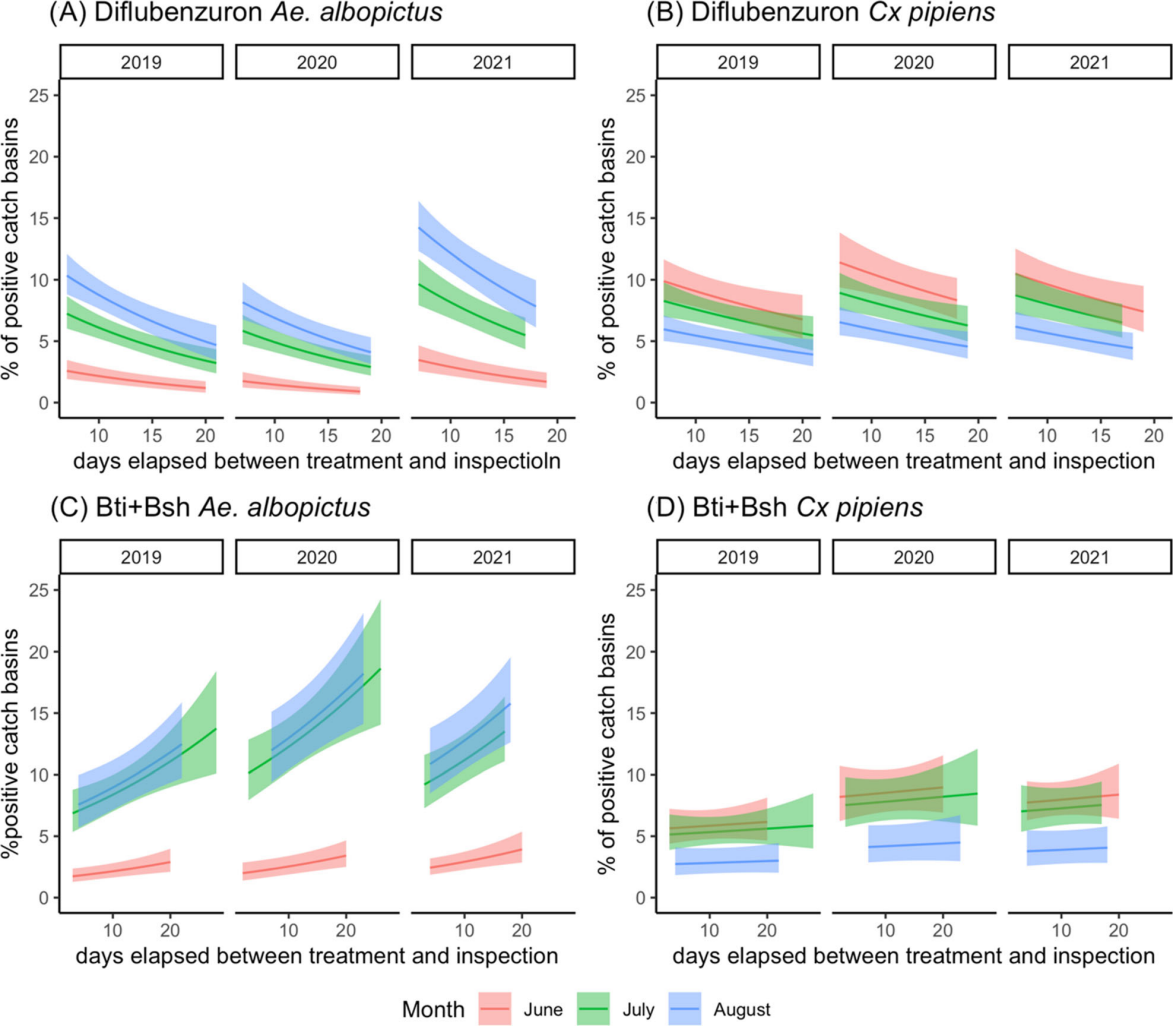
Relationship between the percentage of catch basins containing live mosquito L3–L4 larvae and pupae and the number of days elapsed between treatment and inspection in 2019, 2020 and 2021. (A, B) Estimated percentage of DFB catch basins positive for *Aedes albopictus* (GLM‐1) and *Culex pipiens* (GLM‐2) in a 7–21‐day interval post‐treatment (solid lines). (C, D) Estimated percentage of Bti + Bs‐catch basins positive for *Ae. albopictus* (GLM‐3) and *Cx. pipiens* (GLM‐4) in a 2–28‐day interval post‐treatment (solid lines). Dashed areas indicate 95% CI. Red, June; green, July; blue, August.

Results of GLM‐1 indicate a significant positive association between the percentages of DFB‐treated catch basins positive for *Ae. albopictus* and the number of days that had elapsed between treatment and inspection (DTI). Assuming CRF is at its mean value according to months and years of inspection, positive catch basins decrease from 14% [95% confidence interval (CI) 12%–16%] at 7 days post‐treatment to <1% (95% CI 0.05%–0.09%) at 18 days post‐treatment (Fig. 1(A); Supporting Information, Table S3) A similar pattern is obtained with GLM‐2, with *Cx. pipiens*‐positive catch basins decreasing from 11% (95% CI 9%–13%) at 7 days post‐treatment to 3% (95% CI 2%–5%) at 21 days post‐treatment (Fig. 1(B); Supporting Information, Table S3). In both statistical models, no statistically significant association between the percentage of DFB catch basins positive for *Ae. albopictus*/*Cx. pipiens* and CRF are found (Supporting Information, Fig. S4(A),(B), Table S3).

**Figure 2:**
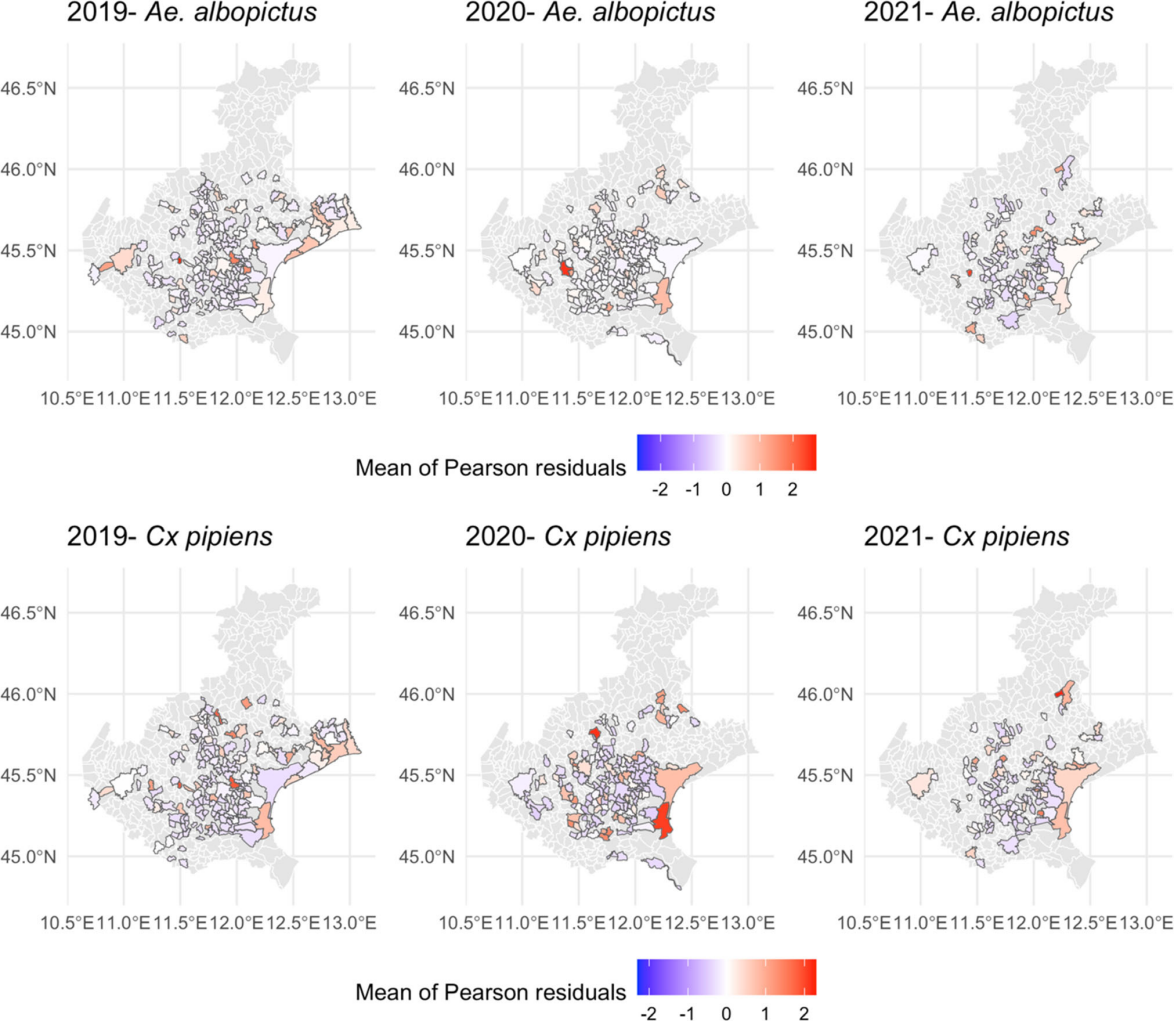
Mean Pearson residuals of diflubenzuron‐treated catch basins for each Veneto municipality for GLM‐1 (*Aedes albopictus*, upper) and GLM‐2 (*Culex pipiens*, lower) in 2019, 2020 and 2021. The legend shows the average of Person residuals in each municipality. Red, statistical model underestimates the observed percentage of positive DFB catch basins; blue, statistical model overestimates the observed percentage of positive DFB catch basins; white, statistical model accurately estimates the observed percentage of positive DFB catch basins; grey, municipality without inspection.

Results from the Pearson residual spatial analysis of GLM‐1 indicate that the statistical model underestimates the percentage of DFB catch basins positive for *Ae. albopictus* (L3, L4 and P) in 9%, 10% and 14% of municipalities in 2019, 2020 and 2021, respectively (Fig. 2). No municipality exhibits consistent underestimation across all three years. The residual analysis of GLM‐2 (*Cx. pipiens*) shows no temporal trend but identifies one municipality (Chioggia) in which underestimation occurred consistently across all three years (Fig. 2).

Sensitivity analysis at the catch basins level shows similar results for GLM‐1 and GLM‐2 when only DFB catch basins with at least one early stage of larvae (L1 and/or L2) are considered (Supporting Information, Table S4).

Among 333 municipalities inspected between 7 and 21 days after DFB treatment (~50 catch basins for each municipality), 24.6% declared that they used DFB for three consecutive years. The results of the sensitivity analyses conducted at the municipality level for both models are consistent with those of the main analyses at the catch basins level (Supporting Information, Table S5).

### 3.2 Outcomes of *Bacillus thuringiensis* var. *israelensis* and *B. sphaericus* treatments at catch basin level

Overall, 10% of Bti + Bs‐treated catch basins with water were positive for mosquito L3, L4 or P, with a higher percentage in 2020 than in the other years (14.2%, $\chi^{2}$ =259.56, *P* < 0.001) (Table 1). A consistent trend is observed for Bti + Bs‐treated catch basins positive for *Ae. albopictus* ($\chi^{2}$ =136,18, *P* < 0.001) and *Cx. pipiens* ($\chi^{2}$ =136,18, *P* < 0.001) (Table 1). The monthly observed percentage of Bti + Bs‐treated catch basins with alive L3, L4 and P *Ae. albopictus* increases from May (1%) to August (7%) every year, whereas *Cx. pipiens*‐positive catch basins follow a similar trend only in 2020 (Supporting Information, Fig. S3 (right)).

The GLM‐2 results indicate a significant negative association between the percentages of Bti + Bs‐treated catch basins positive for *Ae. albopictus* and the number of days that elapsed between treatment and inspection (DTI). Assuming DTI is at its mean value according to the months and years of inspection, the percentage of positive catch basins increases from 1.7% (95% CI 1.2–2) 3 days after treatment to 18% (95% CI 14%–22%) 26 days after treatment (Fig. 1(C); Supporting Information, Table S6). An opposite trend is found for CRF, with the percentage of positive Bti + Bs‐treated catch basins decreasing from 17% (95% CI 15–22 August 2020) with 5 mm CRF to 1% (95% CI 0.9–2% in August 2020) with 120 mm CRF (Supporting Information, Fig. S4(C)). No statistical difference is observed between the percentage of Bti + Bs‐treated catch basins positive for *Cx. pipiens* and either DTI or CRF (Fig. 1(D); Supporting Information, Table S6, Fig. S4(D)).

Results from the Pearson residual spatial analysis of GLM‐3 and GLM4 indicate that the statistical model underestimates the presence of *Ae. albopictus* (L3, L4 and P) in ~10% of municipalities each year. For both species, inspection of the Pearson residual shows no temporal trends, or any municipality where mosquito presence is underestimated across all three years.

Results of the sensitivity analyses show similar results for GLM‐3 and GLM‐4 when only Bti + Bs‐treated catch basins with at least one instar L1–L2 larvae (Supporting Information, Table S7).

At the municipality level, a total of 208 were verified 2 and 28 days after Bti + Bs treatment, with an average of 53 catch basins for each municipality; 17% of these declared that they used Bti + Bs for three consecutive years. Sensitivity analyses conducted at the municipality level for both models reveal no differences compared with analysis at the catch basins level (Supporting Information, Table S8), a statistical association is observed only between DTI and CRF and the presence of *Ae. albopictus* L3, L4 and pupae.

## 4 DISCUSSION

Some regions in Italy have implemented at least 10 years of high‐quality mosquito control management including QA and QC, by coordinated activities involving different actors. Municipalities involve private pest control companies in larval control^24^, ^25^, ^31^ mostly focus on calendar‐based area‐wide larvicidal treatments of rain catch basins in urban and peri‐urban areas. Local health units (ASL) or the municipalities directly use professionals to perform QA and QC. For instance, in Emilia Romagna, treatments are conducted in ~76% of municipalities, with ~16 000 catch basins inspected annually. In addition, ~46% of municipalities performed QC^31^ (www.zanzaratigreonline.it) Similarly, QA and QC approaches are carried out in the neighbouring Veneto region – based on the annual Regional Veneto plan.^24^ These have generated large annual databases, including data on the municipalities involved, larvicides used, treatment schedules, the pest control company performing the treatments, maps of inspected catch basins in public areas and the results of visual inspections after treatments.

This study analysed the QC outcomes of the operational interventions carried out in the Veneto in 2019, 2020 and 2021 focusing on the two main arbovirus vector species (*Ae. albopictus* and *Cx. pipiens*) and on larvicides shown to be most widely used, Bti + Bs and DFB. *Bacillus thuringiensis* var. *israelensis* and *B. sphaericus* bacteria produce crystals composed of multiple protoxins that display specific and potent insecticidal activity against mosquito larvae^14^, ^32^ and show low risk of resistance selection.^30^, ^33^, ^34^, ^35^, ^36^ DFB is a benzoylurea insect growth regulator that inhibits chitin synthesis and shows relatively low acute toxicity to mammals, birds, reptiles, amphibians and fish.^37^ DFB acts during larval development and on the adult's emergence (report annexe^12^). Populations of *Cx. pipiens* resistant to DFB have been reported in north‐eastern Italy.^38^, ^39^, ^40^

Two methodological aspects are relevant to the interpretation of the results. First, according to QC procedures, catch basin inspections were carried out randomly within either 7–21 or 2–28 days after treatment with DFB and Bti + Bs, respectively, limiting the possibility of directly comparing the outcomes of the two treatments. Second, a catch basin was considered positive when at least one live L3, L4 or pupa was collected during inspection, likely introducing the bias of not considering the known effect of DFB on adult emergence.^13^ This choice was inevitable because no data were available on adult emergence from the pupae found in the catch basins.

Overall, results from the inspection of ~13 000 catch basins per year show that most municipalities achieved the objectives of the regional plan (maximum 10% positive catch basins in ~30 catch basins per municipality). In detail, this goal was reached by 69% (*n* = 185 of 241) and 65% (*n* = 116 of 183) of the municipalities using DFB or Bti + Bs, respectively.

Only 9% of the inspected catch basins revealed the presence of live mosquito larvae and/or pupae (*Ae. albopictus*: 5% DFB and 6% Bti + Bs; *Cx. pipiens*: 6% DFB and 5% Bti + Bs). These proportions were generally higher in July and August for *Ae. albopictus* and showed an opposite trend for *Cx. pipiens* (Supporting Information, Fig. S3), reflecting known seasonal dynamics for the two species in Italy.^41^, ^42^, ^43^ The impact of the larval treatments on the adult mosquito population was not directly assessed. However, assuming that in the absence of treatments during the summer season most catch basins would be positive^44^ and represent the major source of mosquitoes in urban sites,^45^ the results suggest that the treatments have contributed in reducing the adult population size, therefore helping in also reducing the risk of transmission of mosquito‐borne diseases. In fact, Guzzetta *et al*.'s^11^ model shows that routine application of larvicides in public catch basins can limit the risk of autochthonous transmission of exotic viruses by *Ae. albopictus* and the size of potential outbreaks.

We specifically assessed the effect of two variables – time elapsed and CRF between treatments and inspections – on the proportion of catch basins positive for *Cx. pipiens* and *Ae. albopictus*, respectively. In the case of Bti + Bs, the model results suggest that the lethal effect is largely maintained over time in the case of *Cx. pipiens* (with an average of 5%–6% of positive catch basins independent of the interval between treatment and inspection). However, in the case of *Ae. albopictus*, the lethal effect decreases over time with an average among months and years of inspection of 2% and 13% positive catch basins 2 and 28 days after treatments, respectively. The opposite pattern is observed for DFB treatments, likely because this IGR requires days after ingestion/contact to show its effect (moulting inhibition).^46^, ^47^ In other words, the result of larval exposure to DFB is delayed. Specifically, the proportion of catch basins positive for *Ae. albopictus* or *Cx. pipiens* is on average 7% and 8%, respectively, at day 7, and 4% for both species at day 21. Notably, a long‐standing effect of DFB was previously observed by Guidi *et al*.,^20^ who reported a decline in *Ae. albopictus* pupae up to 42 days post‐DFB treatment under semi‐field conditions.

In general, the model results do not predict variations in the outcome of the treatment in relation to CRF between treatments and inspection. Furthermore, the *t*‐test result does not indicate a significant difference in CRF between the DFB and Bti + Bs treatments (Supporting Information, Fig. S5) (*t* = −0.613; df = 9378.6, *P* = 0.539). The median CRF values were similar between the two distributions (median of CRF distribution DFB = 21 mm; median of CRF distribution Bti + Bs = 24 mm) However, these results may be affected by a few biases. First, despite the rainfall data being spatially interpolated by weather stations, they likely did not reflect the high heterogeneity at the catch basin level (possibly because of different sizes and positions, other sources of water, or local variation in rainfall). Second, the covariate considered (the CRF between treatment and inspection) does not take into consideration the daily rainfall distribution. However, in the case of *Ae. albopictus*, the model results suggest that the proportion of positive catch basins treated with Bti + Bs is highest in the absence of rain. A similar, but not‐significant trend is observed for DFB‐treated catch basins. This is counterintuitive, as one would expect that rainfall would dilute the larvicide in the catch basins. However, it is likely that the doses used during public treatment are much higher than the lethal ones and that dilution by rainfall is negligible. A possible explanation for the observed trend may be the increased difficulty in collecting larvae by dipping when the quantity of water is larger, particularly at low larval density. The lack of a similar effect on *Cx. pipiens* could be due to an overall higher larval density compared with *Ae. albopictus*, because of different oviposition behaviour (skip oviposition of few eggs per females in *Ae. albopictus* compared with an egg‐raft for *Cx. pipiens*) and/or to the higher adaptation of *Cx. pipiens* to catch basins.^48^

Results of all models were consistent when considering as the response variable the number of positive catch basins out of the total number of catch basins at the municipality level, rather than the presence or absence of mosquito immature stages at catch basin level (as shown above), reinforcing the overall results.

Notably, the modelling analyses resulted in low *R* ^2^ values (Supporting Information, Tables S3 and S6), indicating limited predictive values (small effect size) and suggesting that most of the data variability was not explained by the two chosen covariates. This draws attention to other possible factors – such as operational aspects in larvicide application and physical characteristics of the catch basins (e.g. their structural design, water retention time, or organic matter accumulation, and the presence of other active breeding sites in the vicinity) – which may play a significant role in determining the presence of mosquitoes in treated catch basins. In this respect, we carried out a more in‐depth statistical analysis to identify possible spatial differences in the product's effectiveness. Specifically, analysis of the models’ residuals highlighted areas where catch basin positivity is not explained by the variables considered. This allowed us to pinpoint the municipality in the Venetian lagoon (Chioggia) where the presence of *Cx. pipiens*‐positive catch basins over the three years cannot be consistently attributed to factors such as time of inspection or rainfall. This result can be interpreted in light of findings from a recent study showing the highest frequencies of DFB resistance allele in *Cx. pipiens* populations from the lagoon,^40^ suggesting possible involvement of resistance mechanisms in reducing the effectiveness of larval control. This hypothesis is reinforced by the lack of treatment failure in sympatric *Ae. albopictus* larvae, which may lack known resistance mechanisms.

The following limitations associated with the exploitation of data not collected for experimental purposes but derive from routine QC activities (which are not meant to evaluate the performance of larvicide products) should be considered. First, the study is inherently purely observational and there is a lack of untreated catch basins as a control. Second, the lack of inspection of catch basin pre‐treatment did not allow assessment of the effectiveness of the product used. Third, for feasibility reasons, the sampling effort (the number of dippings per catch basin) was not based on preliminary statistical identification of a threshold of sensibility (e.g. capacity to reveal a defined number of larvae per pupae per litre of water^49^; or adjusted to the quantity of water in the catch basins). Fourth, no data are available on the actual presence/abundance of the two species in the area, hindering our ability to determine whether the absence of mosquitoes in the catch basins is due to treatment effectiveness or lack of infestation. To partially overcome the latter problem, the statistical analysis was restricted to the summer season (June, July and August), when we assumed that larvae/pupae would be found in the catch basins in the absence of treatments. To further reinforce our conclusions, we repeated the modelling efforts on a restricted data set excluding catch basins not containing L1–L2 larvae, assuming that their presence accounts for mosquito infestation in the area during the inspections. Results were consistent with those obtained based on the full data set.

## 5 CONCLUSION

The exploitation of data sets derived from operational QC assessments of mosquito larval control interventions is usually neglected for research purposes, because they lack relevant features, such as the availability of control untreated sites, details on breeding sites (e.g. size, quantity and quality of water, organic load), or number of larvae per site. The results presented here show how high‐quality area‐wide databases from multiple years resulting from QC activities of mosquito control plans may be exploited not only at the local level to evaluate the operational quality, but also for modelling analyses, and may allow us to reach some general conclusions not achievable experimentally and accounting for real‐word heterogeneities. For instance, the results show that whereas the effectiveness of DFB treatments increases in the three weeks following the treatments, the effectiveness of Bti + Bs treatments decreases over time. This suggests that shortening the intervals between Bti–Bs applications may increase control effectiveness, particularly in the case of *Ae. albopictus*, even though testing the product efficacy under control conditions suggests a longer persistence.^50^ Moreover, analysis of the whole data set allowed the identification of a municipality in which treatment failure was consistently not associated with product persistence, or the CRF between treatments. One of the possible reasons for this failure may be the presence of local mosquito populations resistant to the product used. Thus, analysis of area‐wide data from multiple years may help in identifying areas to be prioritized in the monitoring and management of insecticide resistance.

## CONFLICT OF INTEREST

The authors declare no conflict of interest.

## Supporting information


**Data S1.** Supporting Information.

## Data Availability

The data that support the findings of this study are available from the corresponding author upon reasonable request.
