# Supplementary material for: Lessons learned exploiting a multi‐year large‐scale data set derived from operational quality assessment of mosquito larval treatments in rain catch basins
Source: Pest Manag Sci. 2025 Jun 23;81(10):6630–8. doi: 10.1002/ps.70016 (PMC12441763; doi:10.1002/ps.70016)

**Lessons learned exploiting a multi-year large-scale dataset derived from operational quality assessment of mosquito larval treatments in rain catch basins**

Chiara Virgillito^1^, Eleonora Longo^1,2^, Silvia Paolucci^1^, Martina Micocci^1^, Mattia Manica^3^, Federico Filipponi^4^, Stefano Vettore^5^, Davide Bonetto^5^, Andrea Drago^5^, Simone Martini^5^, Alessandra della Torre^1^, Beniamino Caputo^1*^

^1^ -Department of Public Health and Infectious Diseases, Sapienza Università di Roma, Roma, Italy

^2^- Center Agriculture Food Environment, University of Trento, San Michele all’Adige (TN), Italy

^3^ -Center for Health Emergencies, Fondazione Bruno Kessler, Trento, Italy

^4^- National Research Council – Institute for Environmental Geology and Geoengineering (CNR-IGAG), Montelibretti (RM), Italy

^5^ . Entostudio srl, Ponte San Nicolò (PD), Italy

*Corresponding author: beniamino.caputo@uniroma1.it

**Short Running Title**

Lesson learned from mosquito larval control

**Keywords**: larvicide, mosquito, quality control, catch basins

**Figure S1**: Sampling area. a) Topographic map of Veneto region; b) Municipalities where at least one catch basin were inspected in 2019, 2020 and 2021(red).


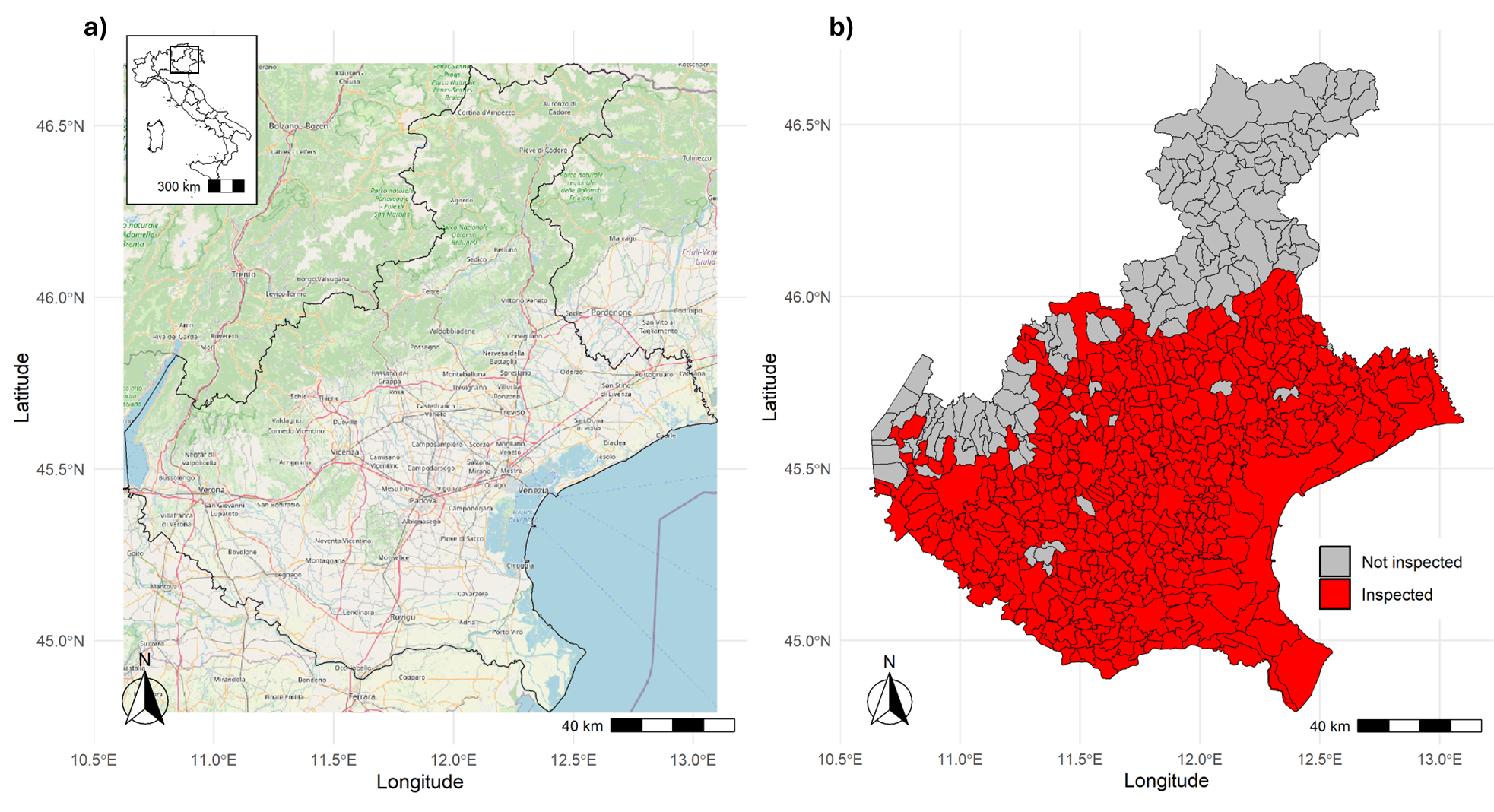


**Table S1**: The 461 municipalities of the Veneto region inspected each year.

| **Municipalities** | **Catch basin inspections** | | |
| --- | --- | --- | --- |
|  | 2019 | 2020 | 2021 |
| Abano Terme | yes | yes | no |
| Adria | yes | yes | yes |
| Agna | yes | yes | yes |
| Agugliaro | yes | yes | yes |
| Albaredo d'Adige | yes | no | no |
| Albettone | no | yes | yes |
| Albignasego | yes | yes | yes |
| Alonte | yes | yes | yes |
| Altavilla Vicentina | yes | yes | yes |
| Altivole | yes | no | yes |
| Angiari | yes | yes | yes |
| Anguillara Veneta | yes | yes | yes |
| Annone Veneto | yes | no | no |
| Arcade | no | yes | yes |
| Arcole | no | no | yes |
| Arcugnano | yes | yes | no |
| Ariano nel Polesine | yes | yes | no |
| Arqua Petrarca | yes | yes | yes |
| Arqua Polesine | yes | yes | no |
| Arre | yes | yes | yes |
| Arsiero | yes | yes | yes |
| Arzergrande | yes | yes | yes |
| Arzignano | yes | yes | yes |
| Asiago | no | yes | no |
| Asigliano Veneto | yes | yes | no |
| Asolo | yes | yes | yes |
| Badia Polesine | yes | yes | no |
| Bagnoli di Sopra | yes | no | no |
| Bagnolo di Po | yes | yes | yes |
| Baone | yes | yes | yes |
| Barbarano Mossano | yes | yes | yes |
| Barbona | yes | yes | yes |
| Bassano del Grappa | yes | yes | yes |
| Battaglia Terme | yes | yes | yes |
| Belfiore | yes | yes | yes |
| Bergantino | yes | yes | yes |
| Bevilacqua | no | yes | yes |
| Boara Pisani | yes | yes | no |
| Bolzano Vicentino | yes | yes | yes |
| Borgo Veneto | no | yes | yes |
| Borgoricco | yes | yes | yes |
| Borso del Grappa | yes | no | no |
| Bosaro | yes | yes | no |
| Bovolenta | yes | yes | yes |
| Bovolone | yes | yes | yes |
| Breganze | yes | yes | no |
| Brendola | yes | no | yes |
| Brogliano | no | yes | yes |
| Brugine | yes | yes | yes |
| Bussolengo | yes | yes | yes |
| Buttapietra | yes | no | yes |
| Cadoneghe | yes | yes | yes |
| Caerano di San Marco | yes | yes | no |
| Caldiero | no | yes | yes |
| Caldogno | yes | yes | yes |
| Calto | yes | yes | yes |
| Caltrano | yes | yes | yes |
| Calvene | no | no | yes |
| Camisano Vicentino | no | yes | yes |
| Campagna Lupia | yes | yes | yes |
| Campiglia dei Berici | yes | yes | yes |
| Campo San Martino | yes | yes | yes |
| Campodarsego | yes | yes | yes |
| Campodoro | yes | yes | yes |
| Campolongo Maggiore | yes | yes | yes |
| Campolongo sul Brenta | yes | no | no |
| Camponogara | yes | yes | yes |
| Camposampiero | yes | yes | yes |
| Canaro | yes | yes | yes |
| Canda | yes | yes | no |
| Candiana | yes | yes | yes |
| Caorle | yes | no | no |
| Cappella Maggiore | no | no | yes |
| Caprino Veronese | no | yes | no |
| Carbonera | yes | no | yes |
| Carceri | yes | yes | yes |
| Carmignano di Brenta | yes | yes | yes |
| Carre | no | no | yes |
| Cartigliano | yes | yes | yes |
| Cartura | yes | yes | yes |
| Casale di Scodosia | no | yes | yes |
| Casale sul Sile | yes | yes | no |
| Casaleone | yes | yes | yes |
| Casalserugo | yes | yes | yes |
| Casier | yes | yes | yes |
| Cassola | yes | yes | yes |
| Castagnaro | yes | no | no |
| Castegnero | yes | yes | yes |
| Castel d'Azzano | yes | no | yes |
| Castelbaldo | yes | yes | yes |
| Castelcucco | yes | yes | yes |
| Castelfranco Veneto | yes | no | no |
| Castelgomberto | yes | no | yes |
| Castelguglielmo | yes | no | yes |
| Castello di Godego | yes | no | yes |
| Castelmassa | yes | yes | yes |
| Castelnovo Bariano | yes | yes | no |
| Castelnuovo del Garda | yes | yes | yes |
| Cavaion Veronese | no | yes | no |
| Cavallino-Treporti | yes | no | no |
| Cavarzere | yes | yes | yes |
| Cavaso del Tomba | no | no | yes |
| Cazzano di Tramigna | no | yes | yes |
| Ceggia | yes | no | no |
| Ceneselli | yes | yes | yes |
| Cerea | yes | no | yes |
| Ceregnano | yes | yes | yes |
| Cervarese Santa Croce | yes | yes | yes |
| Cessalto | yes | yes | yes |
| Chiarano | yes | yes | yes |
| Chioggia | yes | yes | yes |
| Chiuppano | no | yes | no |
| Cimadolmo | yes | yes | no |
| Cinto Caomaggiore | yes | no | no |
| Cinto Euganeo | yes | yes | yes |
| Cismon del Grappa | yes | no | no |
| Cison di Valmarino | no | no | yes |
| Cittadella | yes | yes | yes |
| Codevigo | no | yes | yes |
| Codogne | no | no | yes |
| Codogno | no | yes | no |
| Colceresa | no | yes | yes |
| Colle Umberto | yes | yes | no |
| Cologna Veneta | yes | no | no |
| Colognola ai Colli | yes | no | yes |
| Cona | yes | yes | yes |
| Concamarise | yes | yes | yes |
| Concordia Sagittaria | yes | no | no |
| Conegliano | yes | no | yes |
| Conselve | yes | yes | yes |
| Corbola | yes | yes | yes |
| Cordignano | yes | yes | yes |
| Cornedo Vicentino | yes | no | yes |
| Cornuda | yes | no | no |
| Correzzola | yes | yes | yes |
| Costa di Rovigo | yes | yes | no |
| Costabissara | no | yes | yes |
| Costermano | no | yes | no |
| Creazzo | yes | yes | yes |
| Crespano del Grappa | yes | no | no |
| Crespino | yes | yes | no |
| Crocetta del Montello | no | yes | no |
| Curtarolo | yes | no | yes |
| Dolo | yes | yes | yes |
| Due Carrare | yes | yes | yes |
| Dueville | yes | yes | yes |
| Enego | no | yes | no |
| Eraclea | yes | no | no |
| Erbe | yes | yes | yes |
| Este | yes | yes | yes |
| Farra di Soligo | no | yes | yes |
| Ficarolo | yes | yes | no |
| Fiesso Umbertiano | yes | no | yes |
| Fiesso d'Artico | yes | yes | yes |
| Fontanelle | yes | yes | yes |
| Fontaniva | yes | yes | yes |
| Fonte | yes | yes | yes |
| Fossalta di Piave | yes | no | no |
| Fossalta di Portogruaro | yes | no | no |
| Fosso | yes | yes | yes |
| Frassinelle Polesine | yes | yes | yes |
| Fratta Polesine | yes | no | no |
| Fregona | no | yes | yes |
| Gaiarine | no | yes | no |
| Gaiba | yes | yes | yes |
| Galliera Veneta | yes | yes | yes |
| Galzignano Terme | yes | yes | yes |
| Gambellara | no | yes | no |
| Gambugliano | yes | yes | yes |
| Gavello | yes | yes | yes |
| Gazzo | yes | yes | yes |
| Gazzo Veronese | no | yes | yes |
| Giacciano con Baruchella | yes | yes | no |
| Giavera del Montello | yes | yes | yes |
| Godega di Sant'Urbano | no | yes | yes |
| Gorgo al Monticano | yes | yes | yes |
| Grantorto | yes | yes | yes |
| Granze | yes | yes | no |
| Grisignano di Zocco | yes | yes | yes |
| Gruaro | yes | no | no |
| Grumolo delle Abbadesse | yes | yes | yes |
| Guarda Veneta | yes | yes | yes |
| Illasi | yes | yes | no |
| Isola Rizza | yes | yes | yes |
| Isola Vicentina | yes | yes | yes |
| Isola della Scala | yes | yes | yes |
| Istrana | yes | yes | no |
| Jesolo | yes | no | no |
| Laghi | no | no | yes |
| Lastebasse | no | no | yes |
| Lavagno | yes | yes | yes |
| Legnago | no | no | yes |
| Legnaro | yes | no | yes |
| Lendinara | yes | no | yes |
| Limena | yes | yes | yes |
| Longare | yes | yes | yes |
| Lonigo | no | yes | yes |
| Loreggia | yes | yes | yes |
| Loreo | no | yes | no |
| Loria | no | yes | no |
| Lozzo Atestino | yes | yes | yes |
| Lugo di Vicenza | no | no | yes |
| Lusia | yes | yes | no |
| Lusiana Conco | no | yes | no |
| Malo | yes | yes | no |
| Mansue | no | yes | yes |
| Marano Vicentino | no | yes | no |
| Marcon | yes | yes | yes |
| Mareno di Piave | yes | yes | no |
| Marostica | yes | yes | no |
| Martellago | yes | yes | yes |
| Maser | yes | yes | yes |
| Masera di Padova | yes | no | yes |
| Maserada sul Piave | no | yes | yes |
| Masi | yes | yes | yes |
| Mason Vicentino | yes | no | no |
| Massanzago | yes | yes | yes |
| Meduna di Livenza | no | yes | no |
| Megliadino San Fidenzio | yes | no | no |
| Megliadino San Vitale | yes | yes | yes |
| Melara | yes | yes | no |
| Meolo | yes | no | no |
| Merlara | yes | yes | yes |
| Mestrino | yes | yes | yes |
| Mezzane di Sotto | no | yes | yes |
| Miane | yes | yes | yes |
| Mira | yes | yes | yes |
| Mirano | yes | yes | yes |
| Mogliano Veneto | yes | yes | yes |
| Molvena | yes | no | no |
| Monastier di Treviso | yes | yes | no |
| Monfumo | no | yes | yes |
| Monselice | yes | yes | yes |
| Montagnana | yes | yes | yes |
| Monte di Malo | no | yes | no |
| Montebello Vicentino | no | yes | yes |
| Montebelluna | yes | no | yes |
| Montecchia di Crosara | yes | no | yes |
| Montecchio Maggiore | yes | yes | yes |
| Montecchio Precalcino | yes | yes | yes |
| Monteforte d'Alpone | yes | yes | yes |
| Montegalda | yes | yes | yes |
| Montegaldella | yes | yes | yes |
| Montegrotto Terme | yes | yes | yes |
| Monteviale | yes | yes | yes |
| Monticello Conte Otto | no | yes | yes |
| Montorso Vicentino | no | yes | yes |
| Morgano | yes | yes | yes |
| Moriago della Battaglia | no | yes | yes |
| Motta di Livenza | yes | no | yes |
| Mozzecane | no | yes | no |
| Musile di Piave | yes | no | no |
| Mussolente | yes | yes | yes |
| Nanto | yes | yes | yes |
| Negrar | no | yes | no |
| Nervesa della Battaglia | no | yes | no |
| Noale | yes | yes | yes |
| Nogara | yes | yes | yes |
| Nogarole Rocca | yes | yes | yes |
| Nove | yes | yes | no |
| Noventa Padovana | yes | yes | yes |
| Noventa Vicentina | no | yes | no |
| Noventa di Piave | yes | no | no |
| Occhiobello | yes | yes | yes |
| Oderzo | yes | no | no |
| Oppeano | yes | yes | yes |
| Orgiano | yes | yes | yes |
| Ormelle | yes | yes | no |
| Orsago | no | yes | yes |
| Ospedaletto Euganeo | no | yes | yes |
| Paderno del Grappa | yes | no | no |
| Padova | yes | yes | yes |
| Paese | yes | yes | yes |
| Palu | yes | yes | yes |
| Papozze | yes | yes | yes |
| Pastrengo | yes | no | no |
| Pederobba | no | yes | no |
| Pernumia | yes | yes | yes |
| Peschiera del Garda | no | yes | no |
| Pettorazza Grimani | yes | yes | no |
| Piacenza d'Adige | yes | yes | yes |
| Pianezze | yes | yes | no |
| Pianiga | yes | yes | yes |
| Piazzola sul Brenta | yes | yes | yes |
| Pieve del Grappa | no | yes | yes |
| Pieve di Soligo | no | yes | yes |
| Pincara | yes | yes | yes |
| Piombino Dese | yes | yes | yes |
| Piove di Sacco | yes | yes | yes |
| Piovene Rocchette | yes | yes | no |
| Pojana Maggiore | yes | yes | no |
| Polesella | yes | yes | no |
| Polverara | yes | yes | no |
| Ponso | yes | yes | yes |
| Ponte San Nicolo | yes | yes | no |
| Ponte di Piave | no | yes | yes |
| Pontecchio Polesine | yes | yes | yes |
| Pontelongo | yes | yes | yes |
| Ponzano Veneto | yes | yes | no |
| Porto Tolle | yes | no | yes |
| Porto Viro | no | no | yes |
| Portogruaro | yes | no | no |
| Possagno | no | no | yes |
| Pove del Grappa | yes | yes | yes |
| Povegliano | yes | yes | no |
| Povegliano Veronese | yes | yes | no |
| Pozzoleone | yes | yes | yes |
| Pozzonovo | yes | yes | yes |
| Pramaggiore | yes | no | no |
| Preganziol | yes | yes | yes |
| Quarto d'Altino | yes | yes | yes |
| Quinto Vicentino | no | yes | yes |
| Quinto di Treviso | yes | yes | yes |
| Refrontolo | yes | yes | yes |
| Resana | yes | yes | yes |
| Revine Lago | no | yes | yes |
| Riese Pio X | yes | yes | yes |
| Romano d'Ezzelino | yes | yes | yes |
| Ronca | yes | yes | yes |
| Roncade | yes | no | yes |
| Ronco All'Adige | yes | yes | yes |
| Rosa | yes | yes | yes |
| Rosolina | yes | no | no |
| Rossano Veneto | yes | yes | yes |
| Roverchiara | yes | yes | yes |
| Roveredo di Guè | no | no | yes |
| Rovigo | yes | yes | yes |
| Rovolon | yes | yes | yes |
| Rubano | yes | yes | yes |
| Saccolongo | yes | yes | yes |
| Salara | yes | yes | no |
| Salcedo | yes | no | yes |
| Saletto | yes | no | no |
| Salgareda | yes | no | no |
| Salizzole | yes | yes | yes |
| Salzano | yes | yes | yes |
| San Bellino | yes | yes | no |
| San Biagio di Callalta | no | no | yes |
| San Bonifacio | yes | no | no |
| San Dona di Piave | yes | no | no |
| San Fior | no | yes | yes |
| San Giorgio delle Pertiche | yes | yes | yes |
| San Giorgio in Bosco | yes | yes | yes |
| San Giovanni Lupatoto | no | yes | yes |
| San Martino Buon Albergo | yes | yes | no |
| San Martino di Lupari | yes | yes | yes |
| San Martino di Venezze | yes | yes | no |
| San Michele al Tagliamento | yes | no | no |
| San Nazario | yes | no | no |
| San Pietro Viminario | yes | yes | yes |
| San Pietro di Feletto | no | yes | yes |
| San Pietro di Morubio | yes | yes | yes |
| San Pietro in Cariano | no | no | yes |
| San Pietro in Guè | yes | yes | no |
| San Polo di Piave | yes | yes | no |
| San Stino di Livenza | yes | no | no |
| San Vendemiano | yes | no | no |
| San Vito di Leguzzano | no | yes | no |
| San Zenone degli Ezzelini | no | no | yes |
| Sandrigo | yes | yes | yes |
| Sanguinetto | no | no | yes |
| Sant'Ambrogio di Valpolicella | no | no | yes |
| Sant'Angelo di Piove di Sacco | yes | yes | yes |
| Sant'Elena | yes | yes | no |
| Sant'Urbano | yes | yes | yes |
| Santa Giustina in Colle | yes | yes | yes |
| Santa Lucia di Piave | no | yes | yes |
| Santa Margherita d'Adige | yes | no | no |
| Santa Maria di Sala | yes | yes | yes |
| Santorso | yes | yes | yes |
| Saonara | yes | yes | yes |
| Sarcedo | no | no | yes |
| Sarego | yes | yes | yes |
| Sarmede | yes | no | yes |
| Schiavon | no | yes | yes |
| Schio | yes | yes | yes |
| Scorza | no | yes | no |
| Scorze | yes | no | yes |
| Segusino | yes | no | yes |
| Selvazzano Dentro | yes | yes | no |
| Sernaglia della Battaglia | no | yes | yes |
| Silea | yes | yes | yes |
| Soave | yes | yes | yes |
| Solagna | yes | yes | yes |
| Solesino | yes | yes | no |
| Sommacampagna | yes | no | yes |
| Sona | yes | no | yes |
| Sorga | yes | yes | yes |
| Sossano | no | yes | yes |
| Sovizzo | yes | yes | yes |
| Spinea | yes | yes | yes |
| Spresiano | yes | yes | yes |
| Stanghella | yes | yes | yes |
| Stienta | yes | yes | yes |
| Stra | yes | yes | yes |
| Susegana | no | yes | no |
| Taglio di Po | yes | yes | yes |
| Tarzo | yes | yes | yes |
| Teglio Veneto | yes | no | no |
| Teolo | yes | yes | no |
| Terrassa Padovana | yes | yes | yes |
| Terrazzo | yes | no | yes |
| Tezze sul Brenta | yes | no | yes |
| Thiene | yes | no | no |
| Tombolo | no | yes | yes |
| Tonezza del Cimone | no | no | yes |
| Torre di Mosto | yes | no | no |
| Torreglia | yes | yes | yes |
| Torri di Quartesolo | no | yes | no |
| Trebaseleghe | yes | yes | yes |
| Trecenta | yes | yes | yes |
| Tregnago | yes | no | no |
| Trevenzuolo | yes | yes | yes |
| Treviso | yes | yes | yes |
| Tribano | yes | no | yes |
| Trissino | yes | yes | yes |
| Urbana | yes | yes | yes |
| Val Liona | yes | yes | yes |
| Valbrenta | no | yes | no |
| Valdobbiadene | no | yes | yes |
| Valeggio sul Mincio | yes | yes | yes |
| Valstagna | yes | no | no |
| Vazzola | yes | yes | yes |
| Vedelago | yes | yes | yes |
| Veggiano | yes | yes | no |
| Velo d'Astico | no | yes | no |
| Venezia | yes | yes | yes |
| Verona | yes | yes | yes |
| Veronella | yes | yes | yes |
| Vescovana | yes | yes | yes |
| Vestenanova | no | yes | no |
| Vicenza | yes | yes | yes |
| Vidor | no | yes | yes |
| Vigasio | yes | yes | yes |
| Vighizzolo d'Este | no | yes | yes |
| Vigodarzere | yes | yes | no |
| Vigonovo | yes | yes | yes |
| Vigonza | yes | yes | yes |
| Villa Bartolomea | yes | no | no |
| Villa Estense | yes | no | yes |
| Villa del Conte | yes | yes | yes |
| Villadose | yes | yes | no |
| Villafranca Padovana | yes | yes | yes |
| Villafranca di Verona | yes | no | yes |
| Villamarzana | yes | yes | yes |
| Villanova Marchesana | yes | yes | no |
| Villanova del Ghebbo | yes | yes | no |
| Villanova di Camposampiero | yes | yes | yes |
| Villorba | yes | yes | yes |
| Vittorio Veneto | no | yes | yes |
| Vo | yes | yes | yes |
| Volpago del Montello | yes | no | no |
| Zenson di Piave | yes | yes | no |
| Zermeghedo | no | yes | yes |
| Zero Branco | yes | no | no |
| Zevio | yes | yes | yes |
| Zimella | yes | yes | yes |
| Zovencedo | yes | no | no |
| Zugliano | no | no | yes |

**Figure S2: Average Temperature (red, right y-axis) and Cumulative Rainfall (blue, left y-axis) in the municipalities inspected during the study period (June-August 2019-2021).** Vertical lines= 95% confidence interval for Cumulative Rainfall.


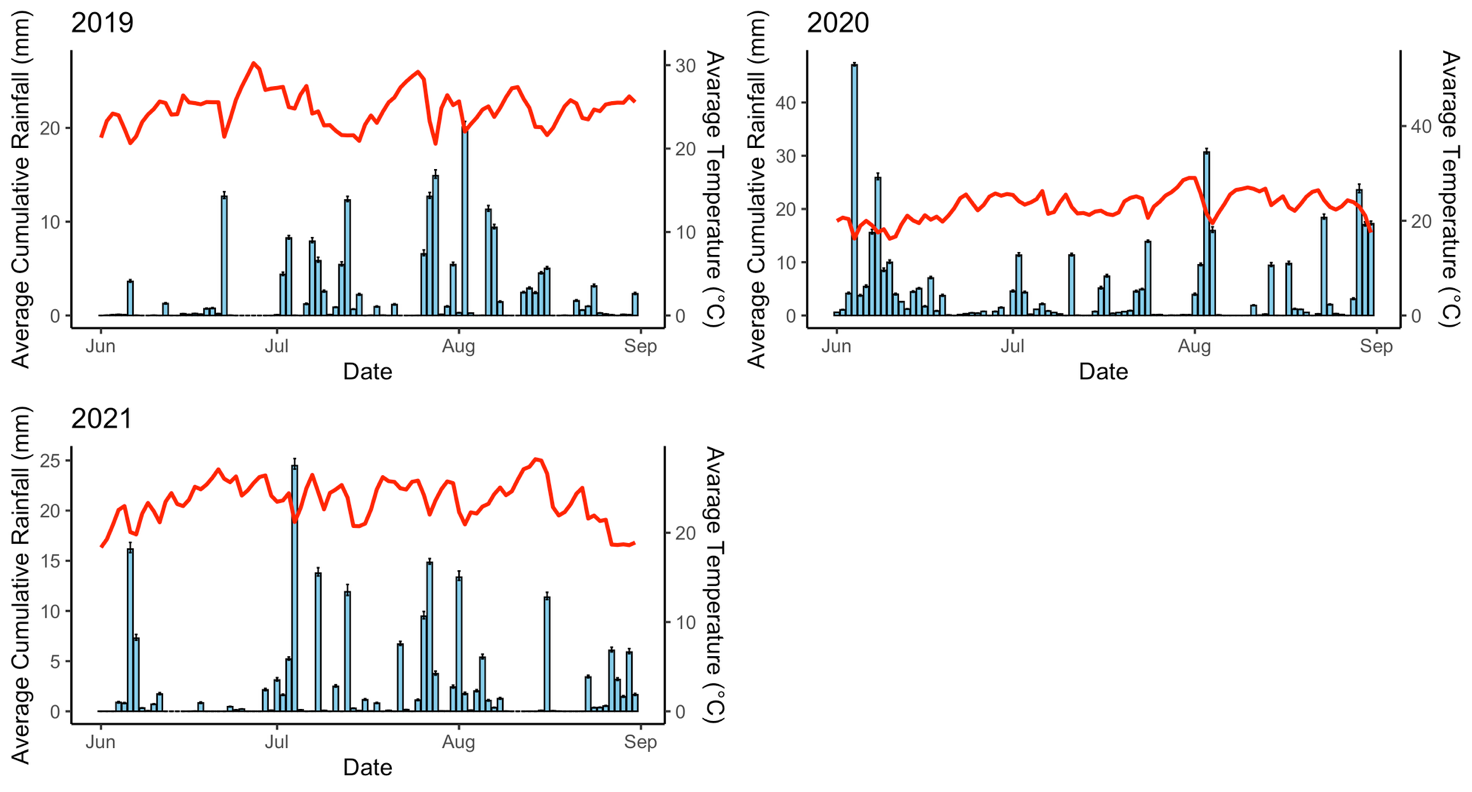


**Table S2: Characteristics of all catch basins inspected in 2019, 2020 and 2021**. *= calculated using dry and non-dry catch basins. **= calculated using non-dry catch basins. DFB= Diflubenzuron; Bti= *Bacillus thuringiensis israelensis*; Bs= *Bacillus sphaericus*

|  | 2019 (N=13,852) | 2020 (N=13,162) | 2021 (N=12,827) |
| --- | --- | --- | --- |
| **Dry catch basins** | 15% (N=2,086) | 17% (N=2,241) | 1% (N=129) |
| **No declaration of larval product*** | 12.5% (N=1,735) | 1,8% (N=233) | 12.4% (N=1,590) |
| **Municipalities (N=560)** | 64.8% (N=363) | 63%(N=353) | 57.7%(N=323) |
| **Products** |  |  |  |
| -Dimethicone | NA | >1% (N=29) | >1% (N=32) |
| -DFB | 54.3% (N=7,524) | 66.7% (N=8,781) | 47.7% (N=6,121) |
| -Bti+Bsh | 24.3% (N=3,371) | 26.6% (N=3,502) | 34.8% (N=4,471) |
| -Pyriproxyfen | >1% (N=75) | 2.4% (N=319) | 1.9% (N=243) |
| -S-Metoprene | >1% (N=31) | >1% (N=48) | >1% (N=28) |
| - DFB + Bti+Bsh | 4.4% (N=611) | 1.6% (N=210) | 2.7% (N=342) |
| - DFB + Pyriproxyfen | >1% (N=64) | >1% (N=40) | NA |
| - DFB + Bti | >1% (N=91) | NA | NA |
| - DFB + Bti + Bti+Bsh | 2.3% (N=321) | NA | NA |
| -Bti + Bti+Bsh | >1% (N=29) | NA | NA |
| **Companies** | NA | 38 | 46 |
| **Catch basins positive for mosquitoes**** (N 2019 = 11,766; N 2020 = 10,921; N 2021 = 12,698) | 9.8% (N=1,152) | 12.9% (N=1,407) | 9.1% (N=1,158) |
| Catch basins positive for *Ae. albopictus*** | 5.9% (N=693) | 7.7% (N=842) | 5.2% (N=663) |
| Catch basins positive for *Cx. pipiens*** | 6.6% (N=780) | 8.5% (N=930) | 5.4% (N=683) |
| **Days between treatment and verification (median)**** | 10 | 12 | 10 |

**Figure S3**: **Observed percentage of catch basins containing live mosquito L3-L4 larvae and pupae in months of collections in 2019, 2020 and 2021** x-axis=months of inspection; y-axis=Percentage of positive DFB-catch basins for *Ae. albopictus* and *Cx. pipiens* (Left) and positive Bti+Bsh -catch basins (Right) in the three years (red=2019; green=2020 and blue=2021). Vertical lines=95% CI of the mean percentage of positive inspected catch basins for each larvicide, years and species.


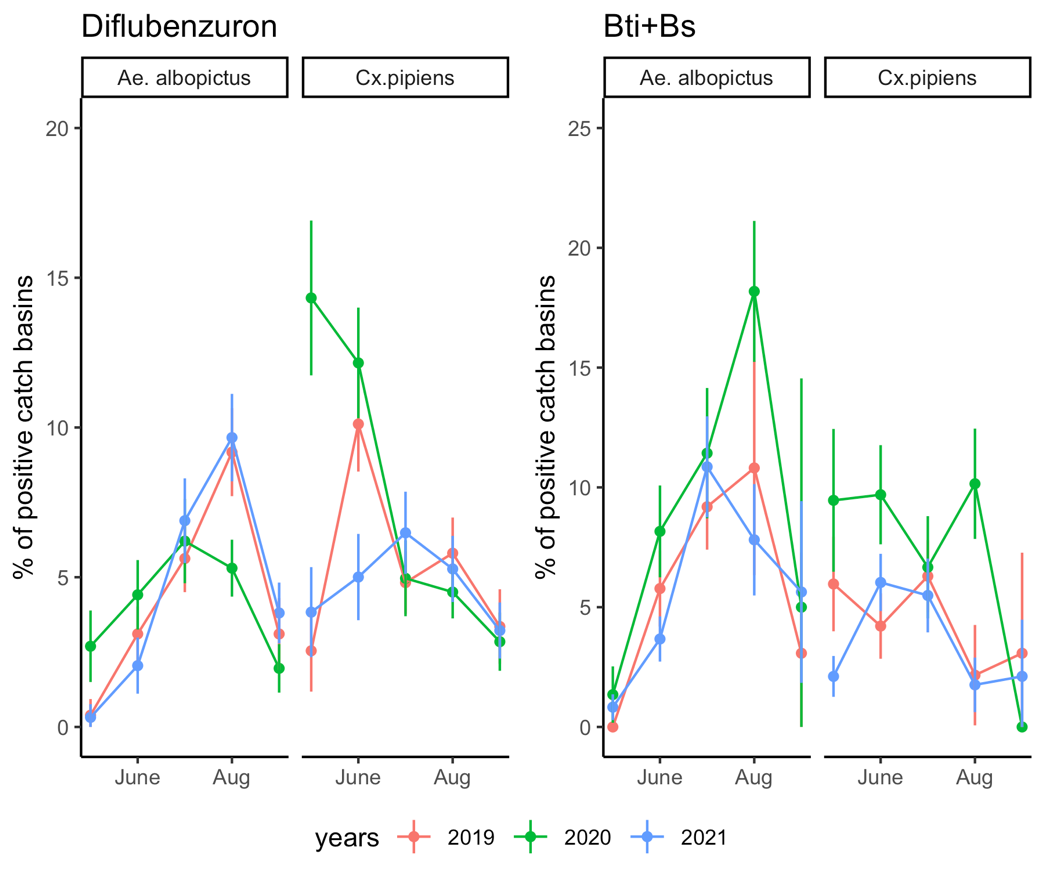


**Table S3**: Summary of *Ae. albopictus* (GLM-1) and *Cx pipiens* (GLM-2) on catch basins with DFB verified between 7 and 21 days after treatment—baseline on intercept=June/2019. * DTI = Days elapsed between treatment and inspection. **= Cumulative rainfall between treatment and inspection. GLM-1 R^2^ =0.04 ; GLM-2 R^2^ =0.07

| **Response variables** | **Coefficient** | **Estimate** | **Std. Error** | **z value** | **Pr(>\|z\|)** |
| --- | --- | --- | --- | --- | --- |
| *Ae. albopictus* | Intercept | -3.141 | 0.195741 | -16.051 | < 2e^-16^ |
|  | DTI* | -0.060979 | 0.013203 | -4.618 | 3.87e-06 |
|  | July | 1.121 | 0.154103 | 7.277 | 3.41e-13 |
|  | August | 1.555 | 0.148744 | 10.457 | < 2e^-16^ |
|  | CRF** | -0.003530 | 0.001894 | -1.864 | 0.06232 |
|  | 2020 | -0.253968 | 0.099833 | -2.544 | 0.01096 |
|  | 2021 | 0.281777 | 0.096988 | 2.905 | 0.00367 |
| *Cx. pipiens* | Intercept | -2.014 | 0.145682 | -13'830 | < 2e^-16^ |
|  | DTI* | -0.031790 | 0.011843 | -2.684 | 0.00727 |
|  | July | -0.214582 | 0.090712 | -2.366 | 0.01800 |
|  | August | -0.585985 | 0.093681 | -6.255 | 3.97e^-10^ |
|  | CRF** | 0.001590 | 0.001521 | 1.045 | 0.29602 |
|  | 2020 | 0.095306 | 0.086067 | 1.107 | 0.26815 |
|  | 2021 | 0.075331 | 0.096020 | 0.785 | 0.43273 |

**Figure S4: Relationship between the percentage of catch basins containing live mosquito L3-L4 larvae and pupae and the cumulative rainfall between treatment and inspection in 2019, 2020 and 2021**. A-B: Estimated percent of DFB-catch basins positive to *Ae. albopictus* (GLM-1) and *Cx pipiens* (GLM-2) as a function of cumulative rainfall between treatment and inspection in 2019, 2020 and 2021 (solid lines). C-D: Estimated percentage of Bti+Bs-catch basins positive to *Ae. albopictus* (GLM-3) and *Cx pipiens* (GLM-4) as a function of cumulative rainfall between treatment and inspection in 2019, 2020 and 2021 (solid lines). Dashed areas=95% of CI. Red=June, green=July, blue=August.

**
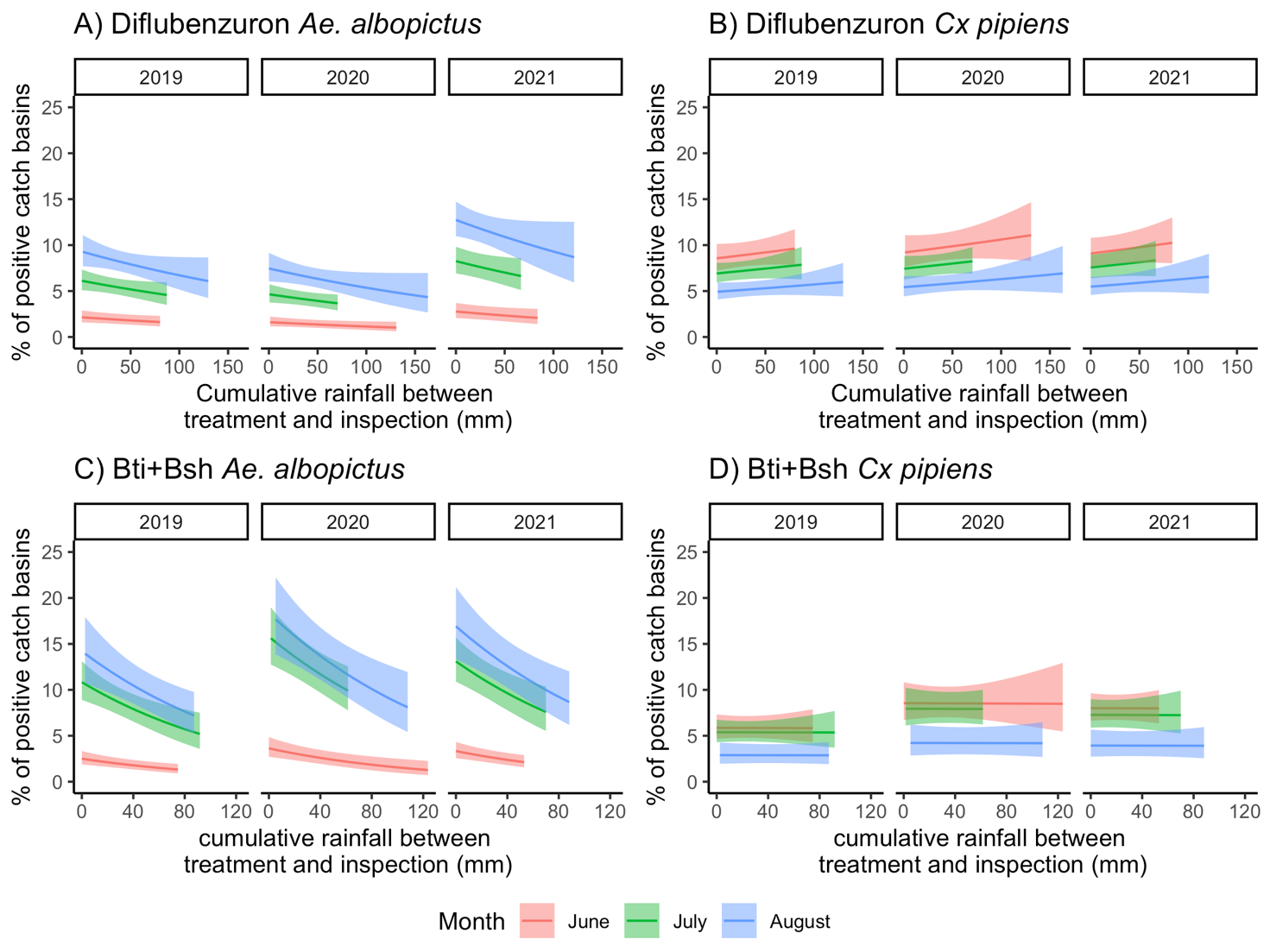
**

**Table S4**: Summary of sensitivity analysis when a subset of the database was considered (only DFB-catch basins where were present at least one instar larvae L1 and/or L2) for *Ae. albopictus* (GLM-1) and *Cx pipiens* (GLM-2) on catch basins with DFB inspected between 7 and 21 days after treatment—baseline on intercept=June/2019. * DTI = Days elapsed between treatment and inspection. **= Cumulative rainfall between treatment and verification.

| **Response variables** | **Coefficient** | **Estimate** | **Std. Error** | **z value** | **Pr(>\|z\|)** |
| --- | --- | --- | --- | --- | --- |
| *Ae. albopictus* | Intercept | 1.087 | 0.6481259 | 1.678 | 0.09336 |
|  | DTI* | -0.0800055 | 0.0389999 | -2.051 | **0.04023** |
|  | July | 0.1682389 | 0.5228034 | 0.322 | 0.74760 |
|  | August | 0.1944260 | 0.4667816 | 0.417 | 0.67703 |
|  | CRF** | -0.0003593 | 0.0056716 | -0.063 | **0.94949** |
|  | 2020 | 0.6508191 | 0.2944900 | 2.210 | 0.02711 |
|  | 2021 | 1.219 | 0.3643161 | 3.346 | 0.00082 |
| *Cx. pipiens* | Intercept | 1.792 | 0.369016 | 4.858 | 1.19e-06 |
|  | DTI* | -0.044949 | 0.029680 | -1.514 | 0.130 |
|  | July | -0.333815 | 0.222939 | -1.497 | 0.134 |
|  | August | -0.082208 | 0.225115 | -0.365 | 0.715 |
|  | CRF** | -0.002847 | 0.003567 | -0.798 | 0.425 |
|  | 2020 | -0.237609 | 0.209148 | -1.136 | 0.256 |
|  | 2021 | -0.314254 | 0.239650 | -1.311 | 0.190 |

**Table S5**: Summary of sensitivity analysis at municipality level for *Ae. albopictus* (GLM-1) and *Cx pipiens* (GLM-2) on catch basins with DFB verified between 7 and 21 days after treatment—baseline on intercept=June/2019. * DTI = Days elapsed between treatment and inspection. **= Cumulative rainfall between treatment and verification.

| **Response variables** | **Coefficient** | **Estimate** | **Std. Error** | **z value** | **Pr(>\|z\|)** |
| --- | --- | --- | --- | --- | --- |
| *Ae. albopictus* | Intercept | -3.521583 | 0.269065 | -13.088 | 2,00E-16 |
|  | DTI * | -0.074533 | 0.018529 | -4.022 | 5.76e-05 |
|  | July | 0.989141 | 0.214360 | 4.614 | 3.94e-06 |
|  | August | 1.639939 | 0.204273 | 8.028 | 9.89e-16 |
|  | CRF** | -0.010108 | 0.002859 | -3.535 | 0.000407 |
|  | 2020 | -0.138574 | 0.134788 | -1.028 | 0.303908 |
|  | 2021 | -0.118945 | 0.130530 | -0.911 | 0.362164 |
| *Cx. pipiens* | Intercept | -2.667497 | 0.205033 | -13.010 | 2,00E-16 |
|  | DTI * | -0.036864 | 0.016894 | -2.182 | 0.05910 |
|  | July | -0.058989 | 0.126215 | -0.467 | 0.64023 |
|  | August | -0.614653 | 0.138209 | -4.447 | 8.7e-06 |
|  | CRF** | -0.002267 | 0.002326 | -0.975 | 0.32956 |
|  | 2020 | 0.150495 | 0.120305 | 1.251 | 0.21095 |
|  | 2021 | -0.364520 | 0.138656 | -2.629 | 0.00856 |

**Table S6.** Summary of *Ae. albopictus* (GLM-3) and *Cx pipiens* (GLM-4) on catch basins with Bti+Bs inspected between 2 and 28 days after treatment—baseline on intercept=June/2019. *DTI= Days elapsed between treatment and inspection. **= Cumulative rainfall between treatment and inspection. GLM-3 R^2^ =0.06; GLM-4 R^2^ =0.1

| **Response variables** | **Coefficient** | **Estimate** | **Std. Error** | **z value** | **Pr(>\|z\|)** |
| --- | --- | --- | --- | --- | --- |
| *Ae. albopictus* | Intercept | -3.953 | 0.171717 | -23.016 | < 2e^-16^ |
|  | DTI* | 0.033 | 0.009758 | 3.391 | 0.000695 |
|  | July | 1.464 | 0.134659 | 10.874 | < 2e^-16^ |
|  | August | 1.649 | 0.152224 | 10.831 | < 2e^-16^ |
|  | CRF** | -0.009 | 0.002515 | -3.426 | 0.000612 |
|  | 2020 | 0.390 | 0.122637 | 3.180 | 0.001472 |
|  | 2021 | -0.022 | 0.119526 | -0.182 | 0.855618 |
| *Cx. pipiens* | Intercept | -2.825 | 0.1517829 | -18.613 | < 2e-16 |
|  | DTI * | 0.009 | 0.0106848 | 0.883 | 0.37744 |
|  | July | -0.122 | 0.1103821 | -1.107 | 0.26835 |
|  | August | -0.837 | 0.1822079 | -4.592 | 4.39e^-06^ |
|  | CRF** | -0.001 | 0.0023341 | -0.252 | 0.80116 |
|  | 2020 | 0.398 | 0.1334996 | 2.985 | 0.00284 |
|  | 2021 | 0.048 | 0.1246544 | 0.389 | 0.69741 |

**Table S7**: Summary of sensitivity analysis when a subset of the database was considered (only Bti+Bs-catch basins where were present at least one instar larvae L1 and/or L2) for *Ae. albopictus* (GLM-3) and *Cx pipiens* (GLM-4) on catch basins with Bti+Bs inspected between 2 and 28 days after treatment—baseline on intercept=June/2019. *DTI= Days elapsed between treatment and inspection. **= Cumulative rainfall between treatment and inspection.

| **Response variables** | **Coefficient** | **Estimate** | **Std. Error** | **z value** | **Pr(>\|z\|)** |
| --- | --- | --- | --- | --- | --- |
| *Ae. albopictus* | Intercept | -0.895241 | 0.349118 | -2.564 | 0.0103 |
|  | DTI* | 0.012414 | 0.022050 | -0.563 | 0.5735 |
|  | July | 1.208091 | 0.251642 | 4.801 | 1.58e-06 |
|  | August | 1.866373 | 0.296105 | 6.303 | 2.92e-10 |
|  | CRF** | -0.011982 | 0.005166 | -2.320 | 0.0204 |
|  | 2020 | 0.406483 | 0.256890 | 1.582 | 0.1136 |
|  | 2021 | -0.120042 | 0.273097 | -0.440 | 0.6603 |
| *Cx. pipiens* | Intercept | 2.515202 | 0.388364 | 6.476 | 9.39e-11 |
|  | DTI * | 0.060609 | 0.022845 | -2.653 | 0.00798 |
|  | July | -1.506004 | 0.255882 | -5.886 | 3.97e-09 |
|  | August | -2.213318 | 0.309866 | -7.143 | 9.14e-13 |
|  | CRF** | -0.002913 | 0.004711 | -0.618 | 0.53640 |
|  | 2020 | -0.894032 | 0.274521 | -3.257 | 0.00113 |
|  | 2021 | -0.571895 | 0.284598 | -2.009 | 0.04449 |

**Table S8:** Summary of sensitivity analysis at municipality level for *Ae. albopictus* (GLM-3) and *Cx*. *pipiens* (GLM-4) on catch basins with Bti+Bs inspected between 2 and 28 days after treatment—baseline on intercept=June/2019. * = Days elapsed between treatment and inspection. **= Cumulative rainfall between treatment and inspection.

| **Response variables** | **Coefficient** | **Estimate** | **Std. Error** | **z value** | **Pr(>\|z\|)** |
| --- | --- | --- | --- | --- | --- |
| *Ae. albopictus* | Intercept | -3.983554 | 0.171106 | -23.281 | 2,00E-16 |
|  | DTI * | 0.034270 | 0.009913 | 3.457 | 0.000546 |
|  | July | 1.476464 | 0.133782 | 11.036 | 2,00E-16 |
|  | August | 1.680545 | 0.151450 | 11.096 | 2,00E-16 |
|  | CRF** | -0.008815 | 0.002523 | -3.493 | 0.000477 |
|  | 2020 | 0.395672 | 0.122021 | 3.243 | 0.001184 |
|  | 2021 | -0.009622 | 0.118852 | -0.081 | 0.935474 |
| *Cx. pipiens* | Intercept | -2.8636419 | 0.1511334 | -18.948 | 2,00E-16 |
|  | DTI * | 0.0092711 | 0.0107815 | 0.860 | 0.38984 |
|  | July | -0.1106357 | 0.1098760 | -1.007 | 0.31398 |
|  | August | -0.8002400 | 0.1802801 | -4.439 | 9.04e-06 |
|  | CRF** | -0.0001602 | 0.0023257 | -0.069 | 0.94507 |
|  | 2020 | 0.4091951 | 0.1327842 | 3.082 | 0.00206 |
|  | 2021 | 0.0705193 | 0.1234513 | 0.571 | 0.56784 |

**Figure S5: Distribution of Cumulative Rainfall between DFB vs Bti+Bs treatment and inspection.** T-student test implemented considering the same time window for both treatment (i.e. 7-day/21-day) reveals no statistical difference between the two treatments (t = -0.613; df=9378.6, p-value=0.539). The upper and lower whisker extends to the highest and lowest values within 1.5*inter-quartile range, respectively. Black dots = outliers, black line= median, the top of the box = first quartile (25^th^ percentile), the bottom of the box = third quartile (75^th^ percentile).


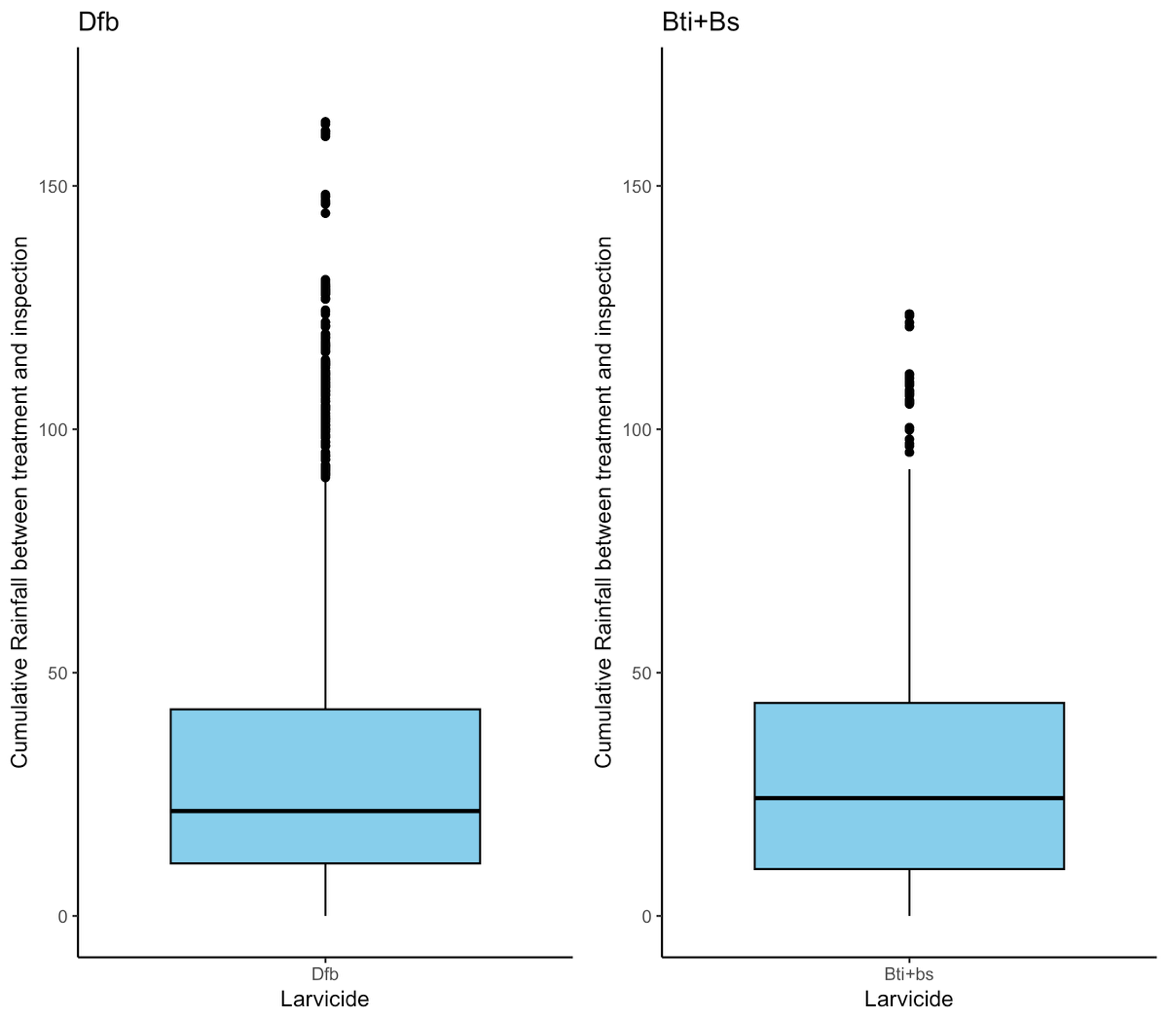

Supplement: Supplementary file 1 — Data S1. Supporting Information. [file PS-81-6630-s001.docx]
